# Global Challenges: Climate Change

**DOI:** 10.1002/gch2.1003

**Published:** 2015-09-21

**Authors:** Georg Feulner

**Affiliations:** ^1^ Earth System Analysis Potsdam Institute for Climate Impact Research Potsdam Germany

## Abstract

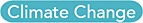

Climate change is arguably the most severe challenge facing our planet during the 21st century. Human interference with the climate system (mainly through the emission of greenhouse gases and changes in land use) has increased the global and annual mean air temperature at the Earth's surface by roughly 0.8 °C since the 19th century (IPCC, 2013). The year 2014 was the hottest one on record so far (NOAA, 2015a), and at the time of writing, 2015 appears to be on track to set a new record (NOAA, 2015b). This trend of increasing temperatures will continue into the future: by 2100, the globe could warm by another 4 °C or so if emissions are not decisively reduced within the next decades (IPCC, 2013). There is broad agreement that a warming of this magnitude would have profound impacts both on the environment and on human societies (IPCC, 2014a), and that climate change mitigation via a transformation to decarbonized economies and societies has to be achieved to prevent the worst of these impacts (IPCC, 2014b).

The spatial and temporal extent of the climate challenge deeply connects it to ethical questions as well. These arise both from the fact that the poorest people on Earth are not significantly contributing to global emissions, but may well feel the impacts most severely, and from the long‐term commitment to future warming and climate change impacts – like sea level or the partial melting of the large ice sheets – which will be felt by future generations. In essence, past and future greenhouse gas emissions seriously affect a large fraction of the still growing human population on our planet and profoundly shape the environment in which our children and grandchildren will have to live in. Humanity therefore has a moral obligation to address the climate challenge. This will have to combine successful negotiations on a binding and effective international climate agreement and bottom‐up initiatives from individuals or communities.

There is a wide range of global threats that certainly require humanity's urgent attention (see the recent report by the World Economic Forum, 2015). These global risks include water, food and energy security, population growth, infectious diseases, and international security, for example. However, climate change is often regarded as one of the most profound global problems. This is mainly due to the sheer scale of climate change impacts – both in terms of its global and temporal spread and of the variety of sectors affected by it – that sets it apart from other planetary challenges. Indeed, recent high‐level initiatives highlight the importance of climate change, including the ground‐breaking encyclical of Pope Francis, the G7 countries' pledge to phase out fossil fuels or Barack Obama's new climate mitigation proposal.

But climate change cannot be considered isolated from other challenges. Indeed, climate change is a truly cross‐cutting issue affecting many sectors and connected to other global challenges. For example, climate change has the potential to impact global water supplies, agricultural production, human health, and our energy infrastructure. In turn, the way in which we produce our energy and food has a profound effect on the Earth's climate system. Finally, the impacts of policies in one of the fields on the other challenges need to be explored if truly sustainable solutions to global problems shall be achieved. These close connections – and the societal and technical challenges of climate mitigation (IPCC 2014b) and adaptation (IPCC 2014a) – require interdisciplinary and transdisciplinary thinking; we hope that our new journal *Global Challenges* can serve as a highly visible forum for research bridging classical scientific disciplines, for ideas which have the potential to directly influence future climate policy and for discussions about new research and different policy options.

Within the climate change focus of *Global Challenges*, we therefore invite submissions related to climate change of the highest quality, with a clear focus on the global view of the climate problem and with relevance for (global) climate policy or bottom‐up initiatives which are a significant step towards a solution of the climate challenge. We explicitly invite submissions connecting climate change to the other challenges covered by the journal. In addition to original research papers, we will regularly commission commentary pieces and review articles highlighting the most relevant recent developments in climate research and policy as well as the most exciting open research questions.

I firmly believe that a journal like *Global Challenges* with its broad scope, its cross‐cutting nature, its focus on policy relevance, and its open‐access publication model is an important and innovative outlet for high‐quality research work on global problems in general. Concerning climate change in particular, I am looking forward to working with the editorial team, the staff at Wiley and the global climate science community to develop *Global Challenges* into one of the major journals in the field.
